# Detection of human activities using multi-layer convolutional neural network

**DOI:** 10.1038/s41598-025-90307-6

**Published:** 2025-02-27

**Authors:** Essam Abdellatef, Rasha M. Al-Makhlasawy, Wafaa A. Shalaby

**Affiliations:** 1 Department of Electrical Engineering, Faculty of Engineering, Sinai University, El-Arish, 45511, Egypt; 2 Electronics Research Institute, Joseph Tito St, El Nozha, P.O. Box: 12622, Cairo, Cairo Egypt; 3 Department of Electronic and Electrical Communication Engineering, Faculty of Electronic Engineering, Menoufia University, Menouf, Egypt

**Keywords:** Human activity recognition, Convolutional neural network, Optimization, Computational biology and bioinformatics, Health care, Engineering

## Abstract

Human Activity Recognition (HAR) plays a critical role in fields such as healthcare, sports, and human-computer interaction. However, achieving high accuracy and robustness remains a challenge, particularly when dealing with noisy sensor data from accelerometers and gyroscopes. This paper introduces HARCNN, a novel approach leveraging Convolutional Neural Networks (CNNs) to extract hierarchical spatial and temporal features from raw sensor data, enhancing activity recognition performance. The HARCNN model is designed with 10 convolutional blocks, referred to as “ConvBlk.” Each block integrates a convolutional layer, a ReLU activation function, and a batch normalization layer. The outputs from specific blocks “ConvBlk_3 and ConvBlk_4,” “ConvBlk_6 and ConvBlk_7,” and “ConvBlk_9 and ConvBlk_10” are fused using a depth concatenation approach. The concatenated outputs are subsequently passed through a 2 × 2 max-pooling layer with a stride of 2 for further processing. The proposed HARCNN framework is evaluated using accuracy, precision, sensitivity, and f-score as key metrics, reflecting the model’s ability to correctly classify and differentiate between human activities. The proposed model’s performance is compared to traditional pre-trained Convolutional Neural Networks (CNNs) and other state-of-the-art techniques. By leveraging advanced feature extraction and optimized learning strategies, the proposed model demonstrates its efficacy in achieving accuracy of 97.87%, 99.12%, 96.58%, and 98.51% for various human activities datasets; UCI-HAR, KU-HAR, WISDM, and HMDB51, respectively. This comparison underscores the model’s robustness, highlighting improvements in minimizing false positives and false negatives, which are crucial for real-world applications where reliable predictions are essential. The experiments were conducted with various window sizes (50ms, 100ms, 200ms, 500ms, 1s, and 2s). The results indicate that the proposed method achieves high accuracy and reliability across these different window sizes, highlighting its ability to adapt to varying temporal granularities without significant loss of performance. This demonstrates the method’s effectiveness and robustness, making it well-suited for deployment in diverse HAR scenarios. Notably, the best results were obtained with a window size of 200ms.

## Introduction

Human Activity Recognition (HAR) systems aim to observe and analyze human activities, as well as accurately interpret ongoing events, as their primary objectives. By understanding human behavior, these systems can evaluate an individual’s performance in daily life and assess their efficiency. The integration of technology has enabled the development of HAR systems that can recognize human activity through various techniques, such as motion analysis, feature extraction, and object recognition. These systems have found applications in a range of industries, including management, athletic training, healthcare, robotics, and entertainment, due to their potential to enhance efficiency and improve overall performance^1^. In the Home Activity Recognition (HAR) task, the primary actions involve sitting, falling, moving, standing, lying down, and the absence of human presence. Of these actions, falling down is particularly relevant to the healthcare and senior care industries, as it is crucial to monitor patients’ movements and activities in these settings to ensure their safety and well-being. The ability to accurately recognize and classify these actions using machine learning algorithms can enable the development of intelligent systems that can detect and respond to patients’ needs in real-time, improving the quality of care and enhancing patient safety^2^.

Conventional surveillance systems rely on human operators to monitor and interpret visual data from various camera viewpoints. However, as the number of camera installations and views increases, the task of human operators becomes increasingly strenuous, leading to decreased productivity levels and higher levels of stress. In response to these challenges, security companies are exploring the use of vision-based technology to automate certain tasks and enhance the accuracy of anomaly detection in camera images^3^.

Various disciplines, such as smart buildings, elderly care, surveillance, and healthcare, are actively pursuing the development of human activity recognition systems. To address this challenge, numerous methods have been proposed, which involve the use of Hidden Markov models and support vector machines to classify activities based on handcrafted features extracted from raw data signals after years of painstaking effort. However, these traditional methods have limitations in terms of performance and efficiency. To overcome these limitations, deep learning techniques are being applied to automatically extract relevant information from the raw data and enhance the overall performance of the activity recognition systems^4^. The detection of actions and diverse human movements through the utilization of data collected from multiple types of sensors is the focus of HAR systems. In addition, HAR has become an essential step in remote health monitoring, assisted living, and human–computer interaction. Convolutional neural networks have gained popularity as they can automatically extract features from time-series data and image or vision data, allowing for the learning of meaningful and high-level features^5,6^. Furthermore, CNNs enhance robustness against noisy accelerometer and gyroscope signals, improving classification accuracy. Compared to traditional machine learning methods, CNNs provide superior generalization, while being computationally more efficient than RNNs and Transformers. The proposed HARCNN model outperforms state-of-the-art approaches, achieving high accuracy across multiple HAR datasets. Its adaptability to real-time applications and mobile deployment highlights its necessity for HAR, particularly in wearable technology and smart environments.

The contributions of this paper are as follows:

I. Developing HARCNN, a CNN model specifically designed to capture intricate spatial and temporal features from raw sensor data, significantly enhancing activity recognition performance.
II. Applying the HARCNN model to multiple datasets to evaluate its efficiency and generalizability in detecting a wide range of human activity recognition (HAR) scenarios.
III. Conducting a detailed comparison of Stochastic Gradient Descent (SGD) and Adaptive Moment Estimation (ADAM), providing insights into their effectiveness in optimizing HAR models for different datasets.
IV. Demonstrating the model’s improved robustness and accuracy for real-time monitoring in critical areas such as healthcare, elderly care, and smart homes, addressing challenges like noisy or incomplete sensor data.
V. Validation of the adaptability of the HARCNN model to dynamic environments, ensuring reliable activity recognition under varied and challenging conditions.
VI. Advancing the potential of the proposed approach to enhance safety, health monitoring, and automation in critical domains such as healthcare, security, and smart environments.

The paper is structured as; Sect. 2 presents the related work, Sect. 3 provides the proposed framework, Sect. 4 discusses the experimental results, and finally the paper is concluded in Sect. 5.

## Related work

The pursuit of comprehending human mobility has been a longstanding objective for scholars from diverse disciplines. This complex phenomenon has been tackled from multiple perspectives, with each field offering a unique approach to understanding its intricacies. A wide range of contributions have been made across various areas, driven by distinct motivations and incentives that have shaped the research agendas of scholars^7^. The primary goal of Human Action Recognition (HAR) is to create a system that can automatically identify and classify a range of predefined actions, such as running, clapping, and other movements, from recorded video sequences. This system has the potential to be highly beneficial in various fields, including human-computer interaction and security video surveillance. The core component of HAR is pattern recognition, which involves utilizing existing knowledge or statistical data to classify the data based on the patterns observed in the video sequences^4^.

Nouriani et al.^8^ propose an algorithm that leverages deep learning to recognize various everyday activities, which is achieved through the integration of a nonlinear observer and inertial sensors. The nonlinear observer utilizes sensor data to estimate body segment tilt angles and sensor bias factors, which are then fed into a deep learning model for activity recognition. This approach enables the algorithm to accurately classify and recognize different everyday activities, such as walking, running, and sitting, among others. The use of inertial sensors and deep learning techniques allows for improved accuracy and efficiency in activity recognition, making this approach a promising solution for various applications in fields such as healthcare and human-computer interaction.

Akter et al.^9^ aimed to enhance the existing Handcrafted Acoustic Representation (HAR) methodology by incorporating Convolutional Neural Networks (CNNs) to provide a more comprehensive feature representation. The proposed approach combined data from multiple convolutional layers and employed an attention mechanism to extract more precise features, resulting in improved model accuracy. The study presents a generalized model structure using Compressed Block Attention Modules (CBAM) and integrates feature combinations from different stages. Instead of relying on complex signal processing methods, the authors utilized spectrograms of raw signals to extract handcrafted features. The performance of the proposed method was evaluated on three datasets: KU-HAR, UCI-HAR, and WISDM. Chen et al.^10^ utilize an extreme learning system to recognize human motion. The study aims to improve the features and classifiers based on available action feature descriptors and conduct experiments on two datasets: the HDM05 motion capture dataset from Bonn University and the MSR-Action3D dataset from Microsoft. Furthermore, the authors propose a novel approach to combine displacement covariance descriptor and direction histogram descriptor to create a new combination that can effectively capture both static and dynamic aspects of joint position information. This study contributes to the advancement of human motion recognition technology and has important implications for applications such as computer vision, robotics, and sports analytics.

Zhang et al.^11^ explore the application of Hybrid Approach to Real time (HAR) in sports competition settings, where a custom-designed sports video-oriented HAR algorithm is developed using a novel multidimensional feature fusion (MFF) technique in conjunction with the kernel extreme learning machine (KELM). This approach aims to provide a more accurate and efficient performance analysis system for sports competitions, which can be used to enhance athlete performance, improve coaching strategies, and provide fans with a more engaging viewing experience. In^12^, the authors investigated the use of various motion data types, including Time-Doppler, Time-Range, and Range-Doppler, as inputs for convolutional neural networks (CNNs) for motion classification. The results of the study revealed that incorporating data from multiple domains significantly improved the accuracy of motion classification, highlighting the importance of considering diverse motion characteristics when developing motion recognition systems. Alawneh et al.^13^ investigate the potential of time series data augmentation to improve the accuracy of deep learning models trained on accelerometer data collected from mobile phones. Specifically, the authors evaluate the performance of three publicly available neural network models - Vanilla, Long-Short Term Memory (LSTM), and Gated Recurrent Units (GRU) - on three different datasets, and examine the effects of two time series data augmentation methods on the models’ accuracy. The findings of this study contribute to the growing body of research on the use of time series data augmentation in deep learning, and provide insights into the potential benefits and limitations of this approach for improving the accuracy of mobile phone-based sensing applications. Butepage et al.^14^ aim to develop a generic representation of human motion that can be learned from a vast corpus of motion capture data and generalize well to unseen motions. To achieve this, they employ an encoding-decoding network that predicts future 3D poses of a human body, allowing them to derive a feature representation of human motion from the most recent past. This approach enables the model to capture the underlying patterns and relationships in human motion, which can be applied to various tasks such as motion synthesis, editing, and recognition. The authors evaluate their method on a large dataset of motion capture recordings and demonstrate its effectiveness in capturing diverse human motions and generalizing to new, unseen motions. Surek et al.^15^ aimed to create sophisticated deep learning models capable of analyzing and categorizing the current human action scenario depicted in videos captured in red, green, and blue colors. This ambitious project involved the development of novel algorithms and techniques to accurately assess and map the complex movements and actions of individuals within these videos. By leveraging the power of deep learning, the researchers sought to provide a comprehensive understanding of the dynamic human action scenario in various contexts, such as sports, workplaces, and social gatherings. The proposed models have the potential to significantly improve the efficiency and accuracy of human action analysis in various fields, including sports analytics, surveillance, and healthcare.

Lin et al.^16^ employ IMU sensor data to prepare for upcoming mobile applications. Lin et al.^17^ suggest using context features in conjunction with a deep model and using background data from several sources to enhance the recognition efficacy. Khan et al.^18^ provide a Wi-Fi CSI-based HAR system that evaluates and compares three deep learning approaches; CNN, long short-term memory (LSTM), and hybrid (LSTM + CNN). Zhu et al.^19^ design a model based on a modified capsule network (MCN). Ravi et al.^20^ present a deep learning-based human activity detection technique to provide an accurate and real-time categorization for wearable low-power devices.

Tang et al.^21^ put forward a deep learning system for recognizing human activities (HAR) with wearable sensors. This system uses multiscale feature learning to enhance accuracy in complicated settings. It merges spatial and temporal data, performing better than older techniques. The method has potential uses in healthcare, smart homes, and industries, demonstrating its reliability and potential for future HAR progress.

The paper by Uddin et al.^22^ presented a deep learning method for recognizing human activities (HAR). This method combines Convolutional Neural Networks (CNN), Convolutional Long Short-Term Memory networks (ConvLSTM), and Long-term Recurrent Convolutional Networks (LRCN). This mixed model captures both spatial and time-related features in video data, which improves the accuracy of activity recognition systems. The authors show how well the model works on common HAR datasets, pointing out its potential uses in areas like surveillance, healthcare, and interaction between humans and computers.

The manuscript authored by Lokesh Lodha^23^ investigated the deployment of deep learning methodologies, specifically Convolutional Neural Networks (CNNs) and Long Short-Term Memory networks (LSTMs), in the identification of human activities. The research underscores the proficiency of these models in effectively capturing spatial and temporal characteristics from sensor data, thereby augmenting the precision of activity recognition systems. The author elaborates on diverse architectures and methodologies, emphasizing their applicability in domains such as healthcare monitoring, fitness tracking, and security surveillance.

The investigation conducted by Yuan et al. addressed the deficiency of extensive labeled datasets in the realm of human activity recognition (HAR) by implementing self-supervised learning (SSL) techniques on the UK Biobank dataset, which encompasses over 700,000 person-days of unlabeled wearable sensor data^24^. The scholars formulated a multi-task SSL architecture that surpassed robust baseline models across seven standard benchmark datasets, attaining an enhancement in F1 score that varied from 2.5 to 100%, with a median enhancement of 18.4%.

The study authored by Kundu et al.^25^ tackles the intricate issue of human activity recognition (HAR) utilizing smartphones, which frequently display discrepancies in sensor measurements attributable to disparate device configurations and holding orientations. The researchers propose a framework grounded in convolutional neural networks (CNNs) that converts sensor data into two-dimensional frequency domain images, thereby proficiently encapsulating temporal patterns and inter-axis spatial characteristics. Furthermore, they present an ensemble of conditional classifiers aimed at augmenting generalization across a spectrum of device configurations and user behaviors.

The method authored by Khazaei et al.^26^ delineates the CDFL framework, a federated learning paradigm meticulously crafted to augment human activity recognition (HAR) while simultaneously addressing critical privacy issues and minimizing communication overhead. CDFL utilizes contrastive learning and deep clustering methodologies to judiciously select representative images that preserve privacy from sensor data, thereby facilitating efficacious model training without necessitating the centralization of data aggregation. This approach alleviates challenges associated with non-independent and identically distributed (Non-IID) data across various devices, which frequently result in protracted convergence times and diminished performance in conventional federated learning architectures. Moreover, Table 1 illustrate a comparison between different studies.

**Table 1:** Comparison between different previous studies.

Paper, year	Dataset	Method	Accuracy
^1^, 2023	UCF50, and another dataset that was created for the experimentation.	Single frame Convolutional Neural Networks (CNNs) and convolutional Long Short-Term Memory	UCF50: 99.80% another dataset:98.93%
^8^, 2022	Inertial sensors	CNN-LSTM	96.47%
^9^, 2023	KU-HAR, UCI-HAR, and WISDM	CNNs	KU-HAR :96.86%, UCI-HAR : 93.48%, WISDM : 93.89%
^13^, 2020	Radar motion-sensing data	CNN	91.5%
^17^, 2023	IMU sensors	Linear Feedforward neural network and a long short-term memory network	93.43%
^16^, 2023	HMDB51	ResNet,	96.7 ± 0.35%
^19^, 2023	Wi-Fi CSI-based HAR system	CNN, LSTM, and LSTM + CNN	LSTM: 95.3% LSTM + CNN: 91.0% CNN: 86.5%
^21^, 2023	UCI-HAR, PAMAP2, WISDM, and UNIMIB-SHAR	New CNN that uses hierarchical-split (HS)	99.02%
^22^, 2024	UCF50 and HMDB51	Convolutional neural network (CNN), convolutional long short-term memory (ConvLSTM), and long-term recurrent convolutional network (LRCN) architectures	96.58%
^23^, 2024	Collected from an accelerometer	Using some tri-axis accelerometers and use of adaptive learning approaches	98%
^25^, 2024	UCI HAR	CNN-based HAR framework that forms 2-D frequency domain images	94%
^26^, 2024	Stanford40, PPMI, and VOC2012	Federated Learning	90.74

## The proposed framework

**Fig. 1:**
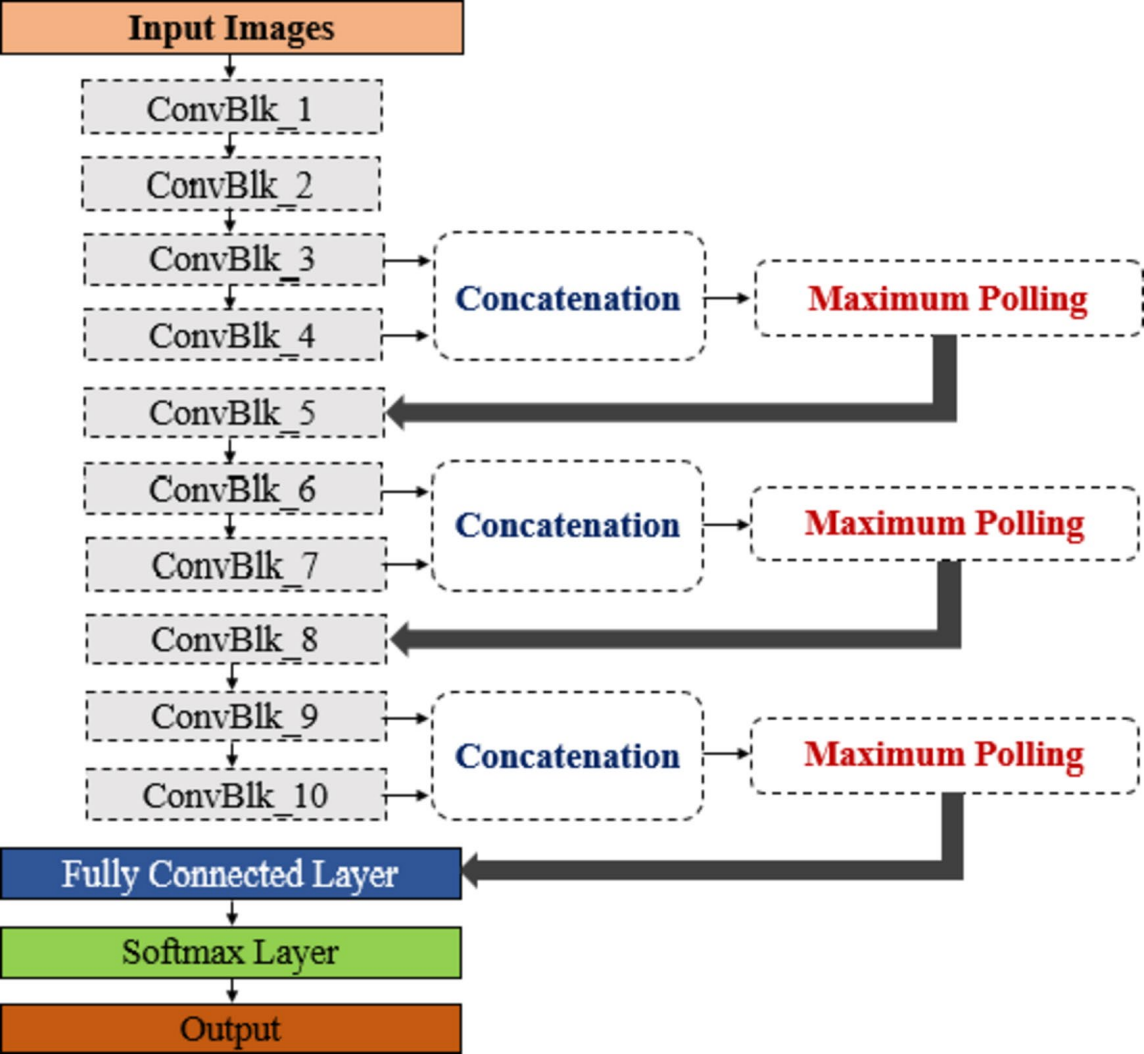
The proposed HARCNN framework.

The proposed framework is designed to leverage the power of Convolutional Neural Networks (CNNs) to improve the accuracy and efficiency of human activity recognition. CNNs have demonstrated remarkable performance in various image and video processing tasks, making them particularly well-suited for analyzing intricate motion patterns and capturing spatial dependencies inherent in human actions. As depicted in Fig. 1, the framework integrates a custom-designed CNN, referred to as the HARCNN model, to perform feature extraction on the input data effectively. The HARCNN model is composed of 10 convolutional blocks, each denoted as “ConvBlk.” Within each block, a ReLU activation function and a batch normalization layer, ensuring stability and enhanced learning capabilities, follow a convolutional layer. A unique aspect of the proposed architecture is the fusion of output features from specific blocks: “ConvBlk_3 and ConvBlk_4,” “ConvBlk_6 and ConvBlk_7,” and “ConvBlk_9 and ConvBlk_10.” These features are integrated using a depth concatenation strategy, enabling the model to combine information from different levels of abstraction. The concatenated features are subsequently passed through a 2 × 2 max pooling layer with a stride of 2, which helps reduce the spatial dimensions while retaining critical information. Furthermore, the detailed specifications and layer sizes of the proposed model are systematically outlined in Table 2, providing insights into the architectural design and computational parameters. This robust configuration underscores the framework’s capability to capture complex patterns in human activities and ensures its adaptability across various recognition tasks.

**Table 2:** Specifications of the HARCNN proposed model.

Layer	Specifications	Size
Input layer	–	224 × 224 × 3
ConvBlk_1	5 × 5 Convolution with 32 filters, stride 2	110 × 110 × 32
ConvBlk_2	3 × 3 Convolution with 48 filters, stride 2	54 × 54 × 48
ConvBlk_3	3 × 3 Convolution with 48 filters, stride 1	52 × 52 × 48
ConvBlk_4	1 × 1 Convolution with 48 filters, stride 1	52 × 52 × 48
Maximum pooling	2 × 2 with stride 2	26 × 26 × 48
ConvBlk_5	3 × 3 Convolution with 64 filters, stride 1	24 × 24 × 64
ConvBlk_6	3 × 3 Convolution with 64 filters, stride 1	22 × 22 × 64
ConvBlk_7	1 × 1 Convolution with 64 filters, stride 1	22 × 22 × 64
ConvBlk_8	3 × 3 Convolution with 128 filters, stride 1	9 × 9 × 128
Maximum pooling	2 × 2 with stride 2	11 × 11 × 64
ConvBlk_9	3 × 3 Convolution with 128 filters, stride 1	7 × 7 × 128
ConvBlk_10	1 × 1 Convolution with 128 filters, stride 1	7 × 7 × 128
Maximum pooling	2 × 2 with stride 2	3 × 3 × 128
Fully connected layer	–	–
Softmax layer	–	–

Convolutional layers produce feature maps through convolving input images with a number of filters as;

1 $$\:F\left(u,v\right)=\sum\:_{m}\sum\:_{n}B\left(u,v\right)C\left(u-m,v-n\right)\:\:\:\:\:\:\:\:\:$$

Where *F*,* B*,* and C* denote the feature maps, input images, and the filter kernel, respectively. The indices of feature maps are denoted by *u* and *v*. The convolutional layers are followed by an activation function and batch normalization layer. Activation functions enable the model to learn complex tasks. Here, we utilize the rectified linear unit (ReLU). Batch normalization layer enhances the stability of CNNs through the normalization of the features. Moreover, it can improve the training speed, minimize the internal covariance, and reduce the problem of overfitting. Pooling layers are used to scale down the spatial size of the generated feature maps, minimize the number of parameters and hence reduce the computational time. Finally, the softmax layer is employed to obtain the class probabilities as follows;

2 $$\:{P\left(y\right)}_{i}=\frac{{e}^{{f}^{T}wi}}{\sum\:_{j=1}^{M}{e}^{{f}^{T}wj}}\:\:\:\:\:\:i=\text{1,2},3,\dots\:.,F\:\:\:\:\:\:\:\:\:\:\:\:\:\:\:\:\:\:\:\:\:$$

Where *P* is the probability, *f* is the feature vector, *T* is the transpose operator, *w* is the weight vector, and *M* is the number of classes. All procedures performed in studies involving human participants were in accordance with the ethical standards of the institutional and/or national research committee and with the 1964 Helsinki declaration and its later amendments or comparable ethical standards.

## Results and discussions

CNN-based HAR systems face challenges such as variability in sensor data, including noise and missing values, which can affect model accuracy and generalization. Additionally, their computational demands may limit deployment on resource-constrained devices like wearable, while over-fitting risks arise from limited or unbalanced datasets. Addressing these issues requires robust data preprocessing, data augmentation, and transfer learning to enhance generalization. Lightweight model architectures and optimization techniques, such as pruning and quantization, can reduce computational overhead. Developing adaptive models for dynamic, real-time data further ensures reliability and scalability, enabling broader application of HAR systems in practical scenarios.

In this section, we outline the experimental configuration and illustrate the results attained by the proposed HARCNN model across the datasets employed in this work^27^. The datasets utilized in this study include the University of California Irvine Human Activity Recognition (UCI HAR), Khulna University Human Activity Recognition (KU-HAR), Wireless Sensor Data Mining (WIDSM), and Human Motion Database (HMDB51). Firstly, we provide concise descriptions outlining the composition of these datasets and their contents. Then evaluating the effectiveness of the proposed model with the datasets using two optimization techniques SGD and ADAM. We assess the output classification performance using metrics such as accuracy, precision, and F-score. Various statistical parameters, including mean, standard error of mean, median, standard deviation, variance, mode, skewness, standard error of skewness, range, minimum, and maximum range, are calculated for each metric. The mean serves as the average value derived from the results. The standard error of the mean quantifies the deviation of the results from the mean, providing a measure of the variability in the data. The median is the central value within the experimental results. The standard deviation and variance values provide insights into the proximity of measurements to the mean value. Lower values of standard deviation and variance are preferred, indicating greater consistency and less dispersion in the data around the mean. The mode represents the value that appears most frequently in the experiments. Correct skewness indeed measures the degree of symmetry or asymmetry in the distribution of data. Positive skewness indicates a longer or fatter tail on the right side of the distribution, while negative skewness suggests a longer or fatter tail on the left side. A distribution is considered highly skewed when skewness is less than − 1 or greater than one, moderately skewed when skewness falls between − 1 and − 0.5 or between 0.5 and 1, and approximately symmetric when skewness is between − 0.5 and 0.5. The standard error of skewness is the ratio of skewness to the standard error, while the range represents the difference between the maximum and minimum values in the dataset.

### Performance of proposed model using the UCI HAR dataset

The UCI HAR (Human Activity Recognition) dataset is indeed a well-known benchmark dataset in the field of activity recognition. Researchers at the University of California, Irvine (UCI), created the dataset, it is widely used for developing, and evaluating algorithms for recognizing human activities based on sensor data. It includes 10,299 images of six different actions for 30 participants, aged ranged from 19 to 48.The UCI-Dataset is the inaugural dataset captured using embedded tri-axial sensors, including accelerometers and gyroscopes, within a smartphone (Samsung Galaxy S II) positioned on the waist of 30 subjects engaged in six different daily activities as shown in Table 3.

**Table 3:** UCI-HAR dataset activities.

Class	Activity
Walking	Participant walks horizontally forward in a direct position
Walking (upstairs)	Participant walks upstairs
Walking (downstairs)	Participant walks downstairs
Sitting	Participant sits on a chair
Standing	Participant stands inactive
Laying	Participant sleeps or lies down

Table 4 presents the achieved accuracy, precision, and F-score during testing phases on subsets of the UCI HAR dataset, employing various optimization techniques and learning rate values. The peak accuracy on the testing subset, nearly 96%, was realized with a learning rate set at 0.0001, utilizing the Stochastic Gradient Descent (SGD) optimization technique. The precision and F-score consistently fall within the range of 95% under similar conditions. Statistical analysis reveals that the mean accuracy and precision across multiple attempts approximate 94%, while the mean F-score stands at 93%. The median values indicate that accuracy hovers around 94%, while the median for the F-score is approximately 93%. The standard deviation is approximately 0.89 for accuracy, and 1.5 for both precision and F-score, indicating variability around the mean values in the dataset. Additionally, the variance is approximately 0.8 for accuracy, and 2.3 for both precision and F-score, providing further insight into the spread of data points from the mean in each respective metric. The skewness values indicate a slight positive skewness of 0.4 for accuracy, and a moderate negative skewness of -0.61 for both precision and F-score. This suggests a tendency for the data to be slightly skewed towards higher accuracy values and lower precision and F-score values. The dataset displays a range of approximately 3 for accuracy, and 5 for precision and F-score, indicating the span between the maximum and minimum values in each respective metric.

**Table 4:** Performance of the proposed model for UCI-HAR dataset.

Optimizer	#Run	LR	Accuracy	Precision	F-score
SGD	Run (1)	0.01	95.23	95.81	95.3
	Run (2)	0.001	93.53	93.28	92.7
	Run (3)	0.0001	96.22	95.93	95.34
	Run (4)	0.00001	93.75	94.48	94.89
	Run (5)	0.000001	94.79	95.13	94.51
ADAM	Run (6)	0.01	94.48	92.78	93.15
	Run (7)	0.001	93.01	91.33	90.7
	Run (8)	0.0001	94.41	92.81	92.17
	Run (9)	0.00001	94.45	95.02	94.33
	Run (10)	0.000001	94.45	94.94	93.25
Statistical analysis					
Mean			94.4320	94.1510	93.6340
Standard error of mean			0.28414	0.48023	0.47751
Median			94.4500	94.7100	93.7900
Standard deviation			0.89853	1.51863	1.51001
Variance			0.807	2.306	2.280
Skewness			0.449	-0.626	-0.678
Standard error of skewness			0.687	0.687	0.687
Range			3.21	4.60	4.64
Minimum			93.01	91.33	90.70
Maximum			96.22	95.93	95.34

**Fig. 2:**
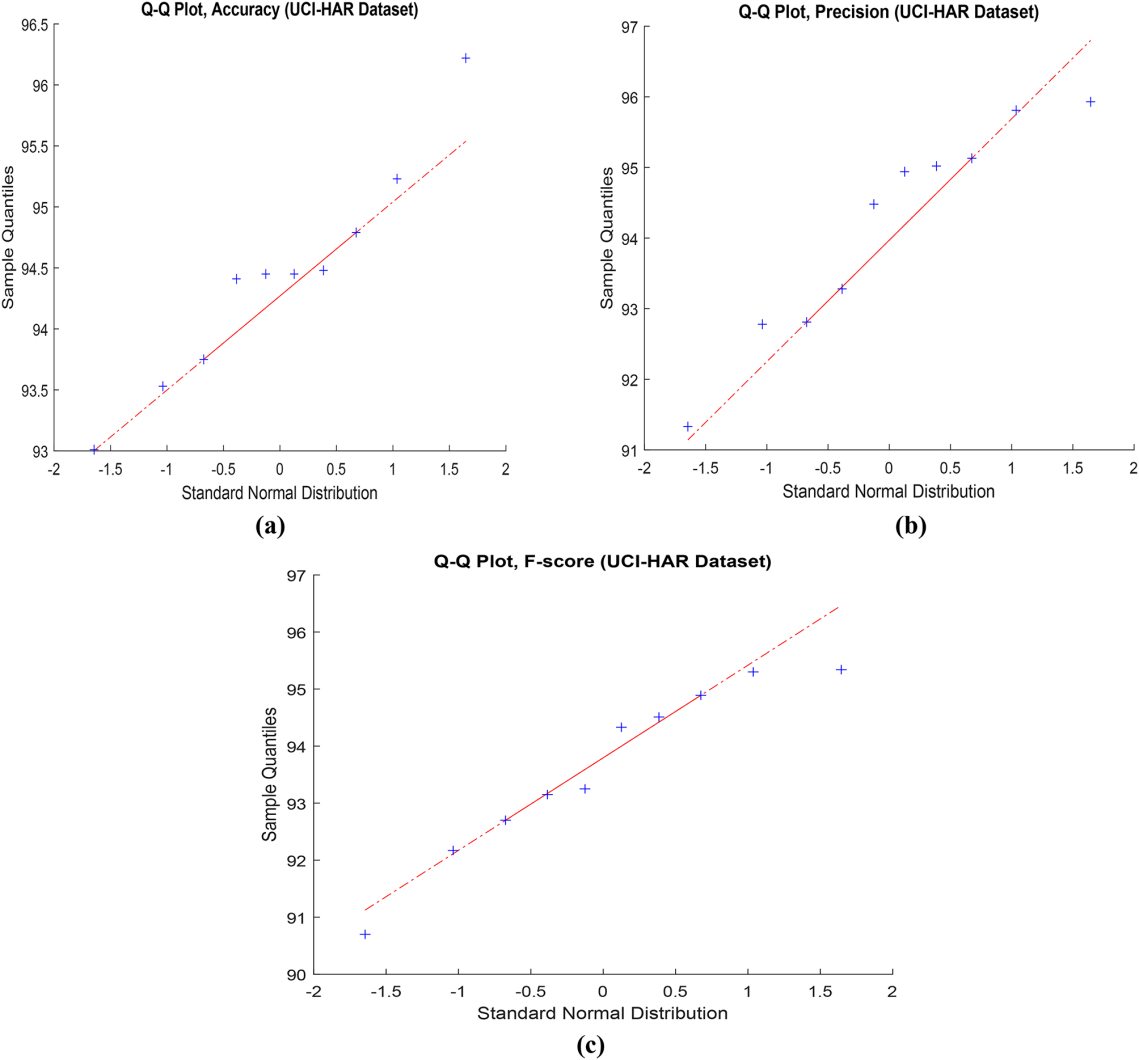
Q-Q Plot for accuracy, precision, and F-score in the proposed framework using UCI-HAR dataset.

**Fig. 3:**
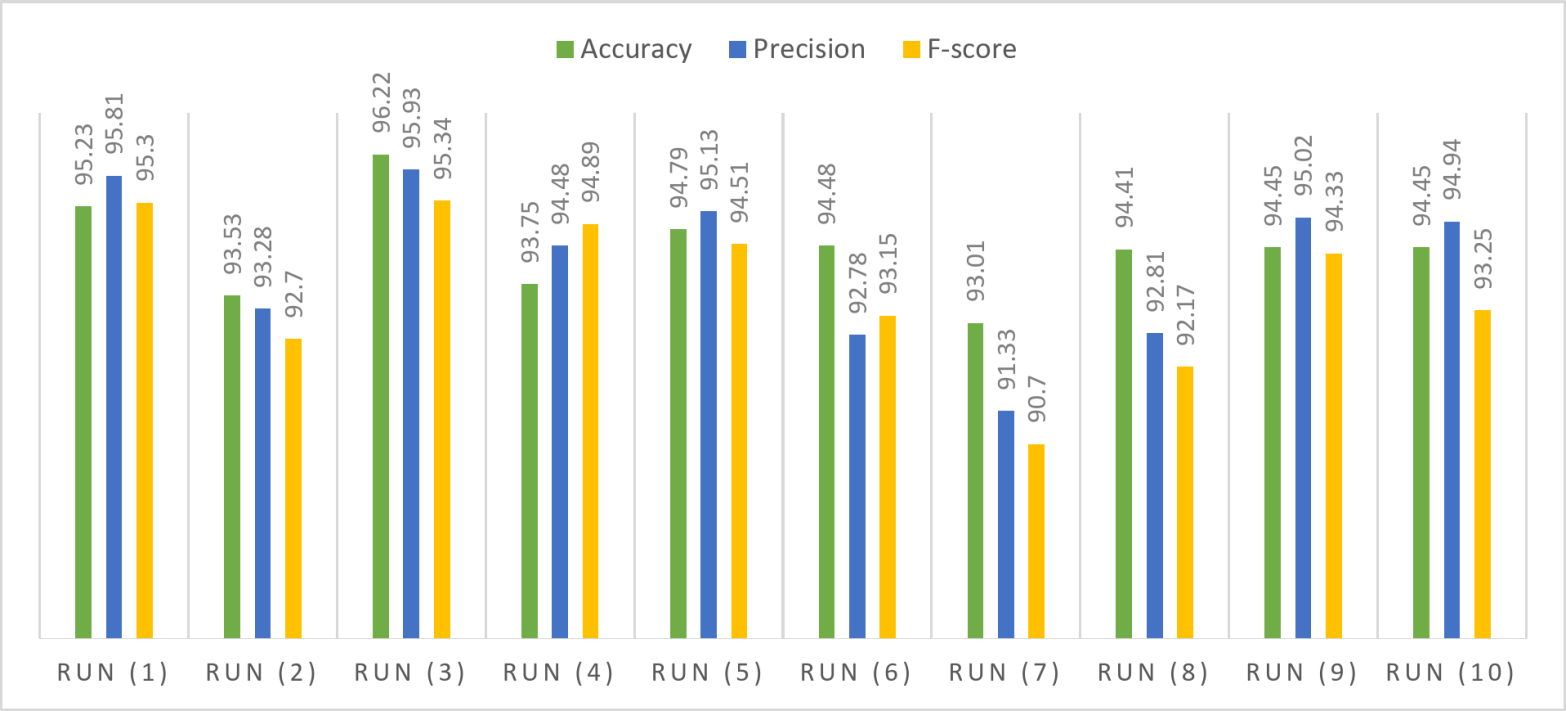
Performance assessment of the proposed model on UCI-HAR dataset across varied hyper-parameters.

**Fig. 4:**
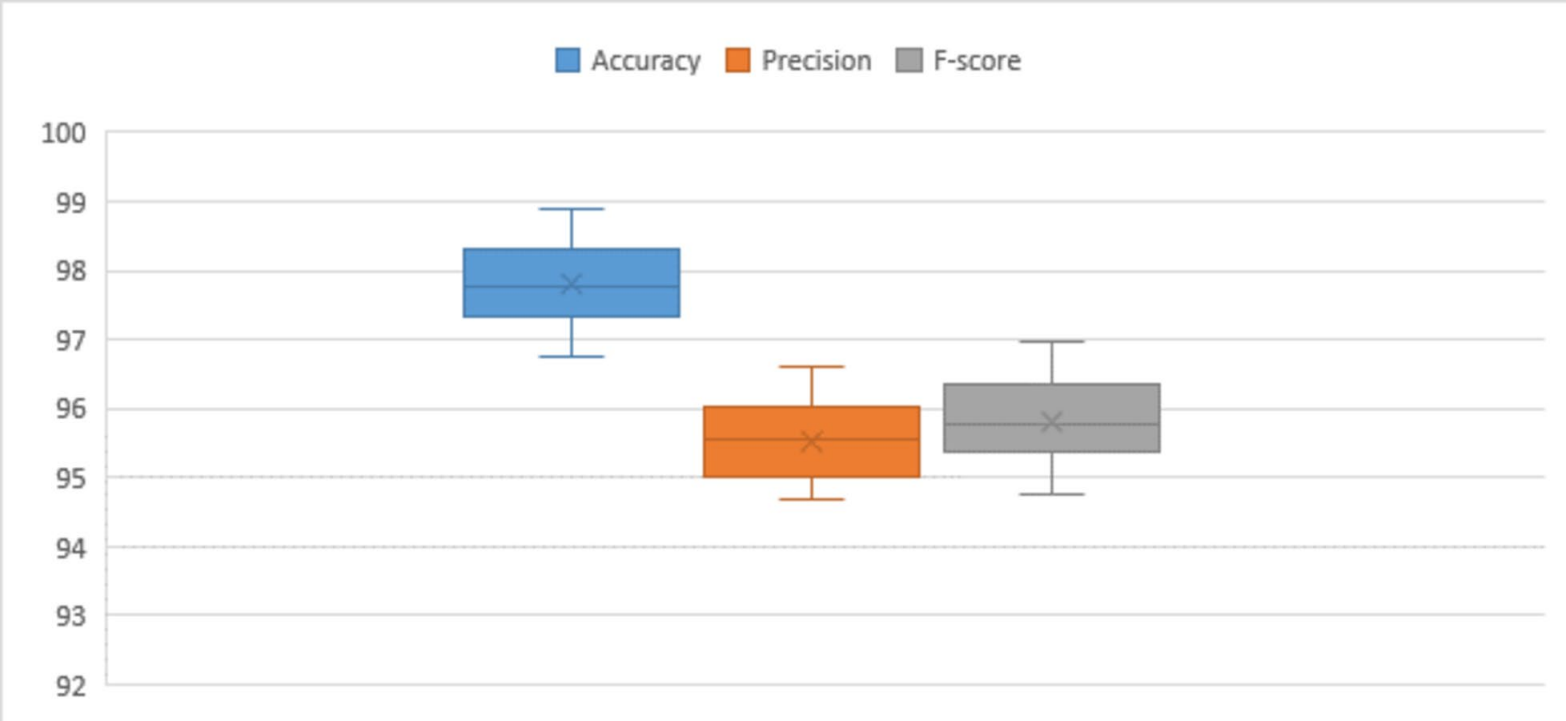
Visual representation of the proposed framework performance utilizing UCI-HAR dataset.

Figure 2 illustrates the Quantile-Quantile (Q-Q) analysis for key performance metrics, namely accuracy, precision, and F-score, in the context of the proposed framework. The data utilized for this evaluation is sourced from the UCI-HAR dataset. The Q-Q plot is a valuable tool for comparing the quantiles of the observed distribution of these metrics against the quantiles of an expected theoretical distribution. Figure 3 illustrates the evaluation of the proposed model’s performance on the UCI-HAR dataset under different hyper-parameter settings. The analysis provides insights into how changes in hyper-parameters influence the model’s overall effectiveness on the given dataset. Figure 4 depicts a visual overview of the proposed framework applied to the UCI-HAR dataset. The illustration aims to provide a clear and concise representation of the key components and interactions within the proposed framework.

### Performance of proposed model using the KU-HAR dataset

The KU-HAR dataset, released in the early months of 2021, the work authored by Nahid^28^ and colleagues. It accessible online, encompasses three versions of the collected HAR data. Two of these variants preserve the raw time-domain signals in their original form, directly acquired from participants through accelerometer and gyroscope sensors. The third variant comprises sub samples extracted from the initial samples. The samples were subsequently divided into 20,750 images for 90 participants, aged between 18 and 34. Table 5 provides KU-HAR dataset activities.

**Table 5:** KU-HAR dataset activities.

Class	Activity
Stand	Standing still on the floor
Sit	Sitting still on a chair
Talk–sit	Talking with hand movements while sitting on a chair
Talk–stand	Talking with hand movements while standing up or sometimes walking around within a small area
Stand–sit	Repeatedly standing up and sitting down
Lay	Laying still on a plain surface (a table)
Lay–stand	Repeatedly standing up and laying down
Pick	Picking up an object from the floor by bending down
Jump	Jumping repeatedly on a spot
Push-up	Performing full push-ups with a wide-hand position
Sit-up	Performing sit-ups with straight legs on a plain surface
Walk	Walking 20 m at a normal pace
Walk backward	Walking backward for 20 m at a normal pace
Walk-circle	Walking at a normal pace along a circular path
Run	Running 20 m at a high speed
Stair-up	Ascending on a set of stairs at a normal pace
Stair-down	Descending from a set of stairs at a normal pace
Table tennis	Playing table tennis

In Table 6, the obtained accuracy, precision, and F-score during testing phases on subsets of the KU-HAR dataset are showcased. The highest accuracy achieved on the testing subset, reaching almost 99%, was attained with a learning rate set at 0.00001, employing the SGD optimization technique. Precision and F-score consistently remain within the range of 97% under comparable conditions. Through statistical analysis, it has been determined that the average F-score and precision over multiple attempts approximate 96%, while the average accuracy stands at 98%. The median values point to a sustained accuracy level of approximately 98%, while both precision and F-score maintain a median of around 96%. The standard deviation, at approximately 0.6 for accuracy, precision, and F-score, signifies a degree of variability around the mean values in the dataset. Furthermore, a variance is around 0.4, the data exhibits a moderate degree of dispersion from the mean values, suggesting some variability in the dataset. The skewness values indicate a minor positive skewness of approximately 0.2 for accuracy, precision, and F-score. The range in the dataset is approximately 2, representing the difference between the maximum and minimum values.

**Table 6:** Performance of the proposed model for KU-HAR dataset.

Optimizer	#Run	LR	Accuracy	Precision	F-score
SGD	Run (1)	0.01	97.34	95.49	95.54
	Run (2)	0.001	96.76	94.67	94.74
	Run (3)	0.0001	98	95.64	95.97
	Run (4)	0.00001	98.91	96.61	96.97
	Run (5)	0.000001	97.47	95.18	95.47
ADAM	Run (6)	0.01	97.55	95.07	95.44
	Run (7)	0.001	97.98	95.73	96.03
	Run (8)	0.0001	97.31	94.78	95.17
	Run (9)	0.00001	98.5	96.18	96.46
	Run (10)	0.000001	98.26	95.99	96.34
Statistical analysis					
Mean			97.8080	95.5340	95.8130
Standard error of mean			0.20304	0.19693	0.21089
Median			97.7650	95.5650	95.7550
Standard deviation			0.64206	0.62274	0.66690
Variance			0.412	0.388	0.445
Skewness			0.179	0.231	0.172
Standard error of skewness			0.687	0.687	0.687
Range			2.15	1.94	2.23
Minimum			96.76	94.67	94.74
Maximum			98.91	96.61	96.97

**Fig. 5:**
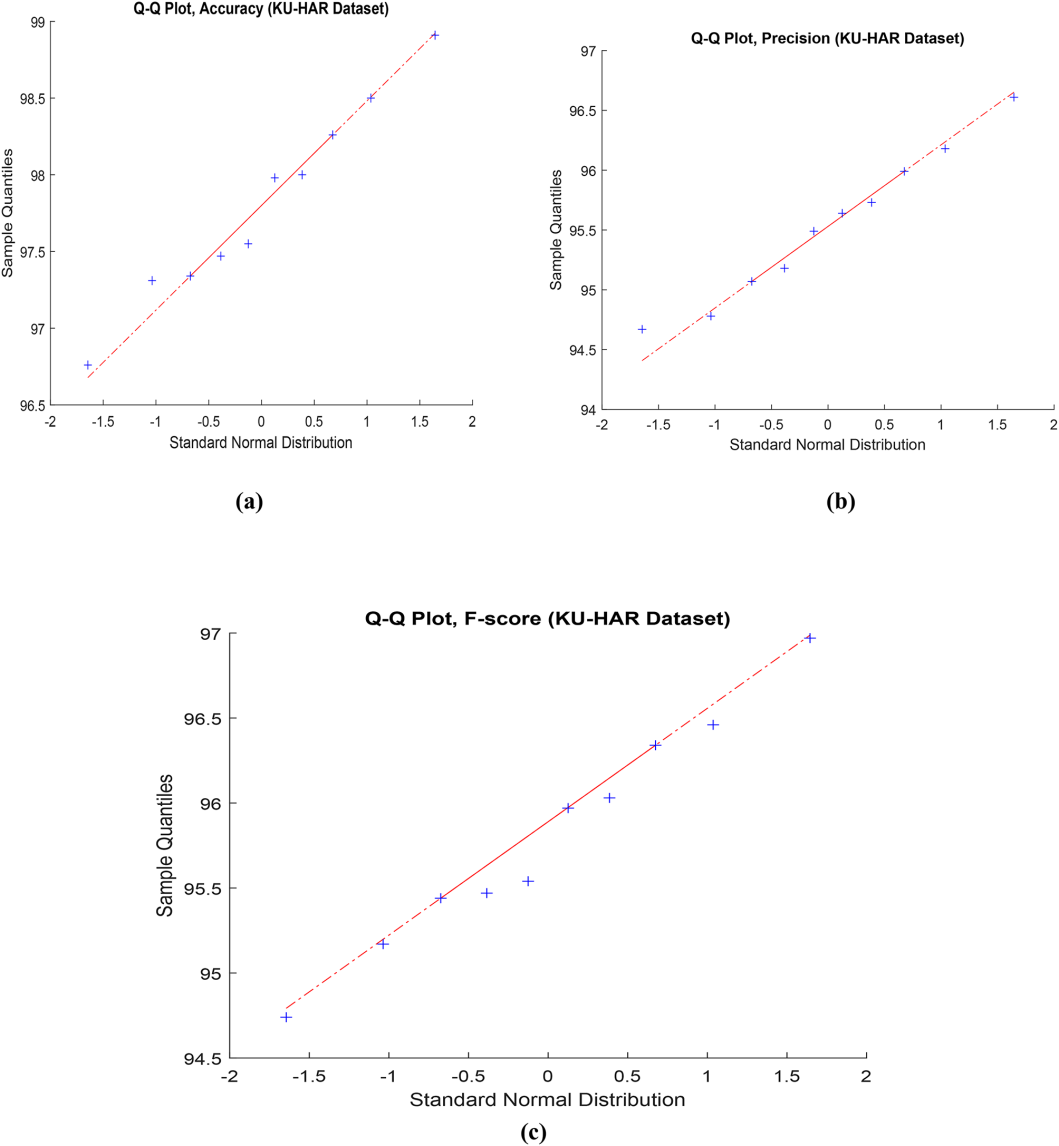
Q-Q Plot for accuracy, precision, and f-score in the proposed framework using KU-HAR dataset.

**Fig. 6:**
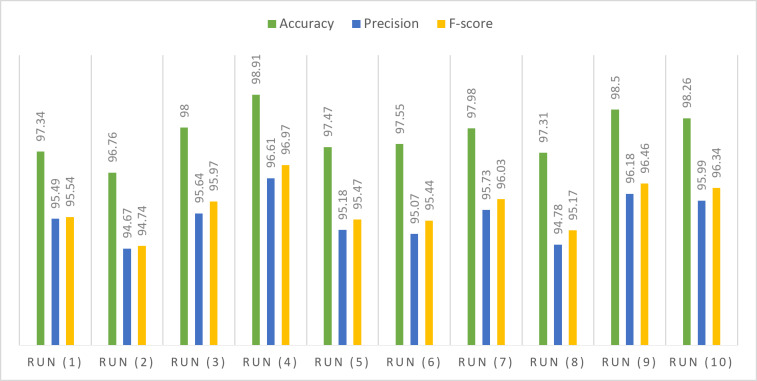
Performance assessment of the proposed model on KU-HAR dataset across varied hyper-parameters.

**Fig. 7:**
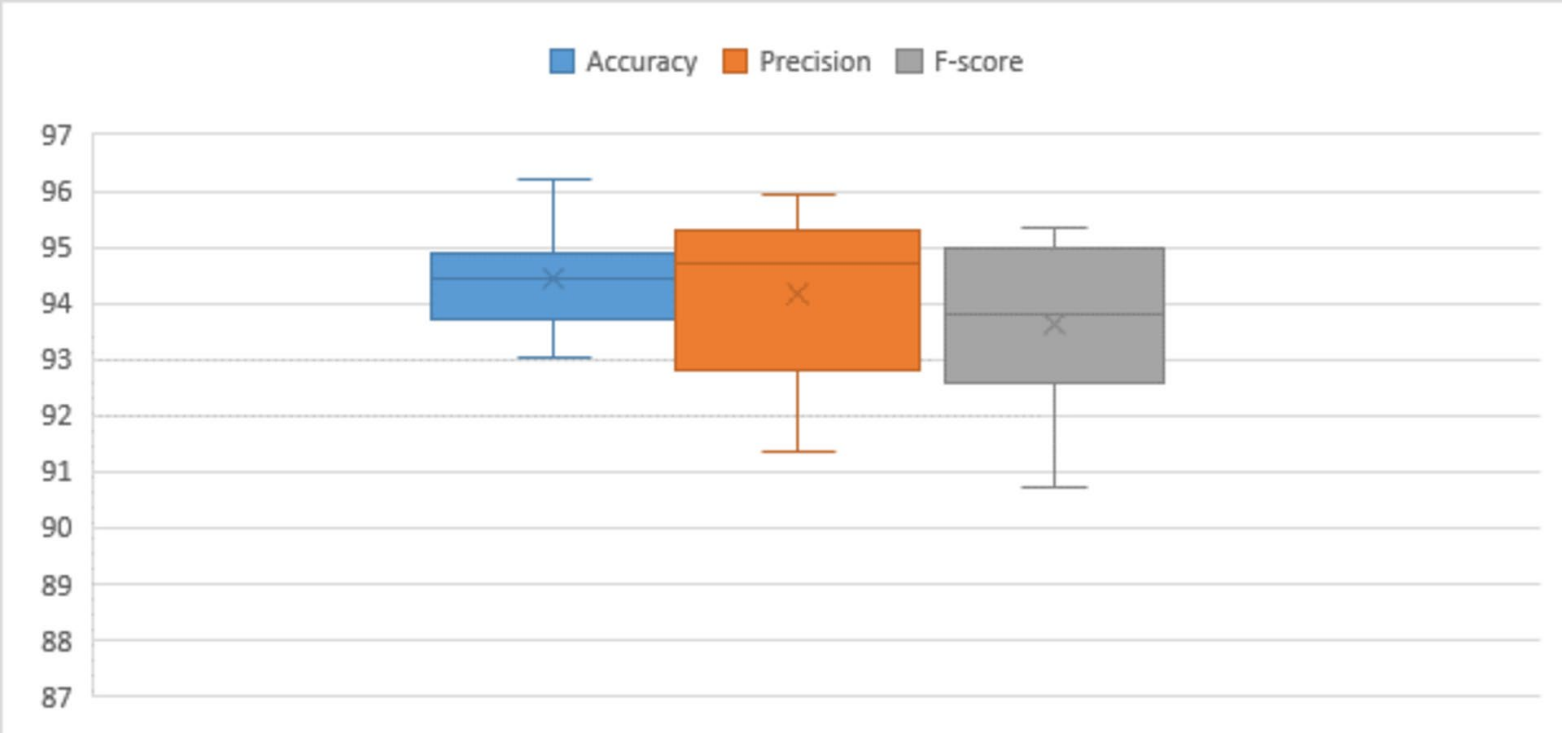
Visual representation of the proposed framework performance utilizing KU-HAR.

Figure 5 shows the (Q-Q) statistical technique that used to compare the distribution of observed data with the expected distribution. In the context of key performance metrics like accuracy, precision, and F-score, Q-Q analysis can help assess the goodness of fit between the proposed framework’s predicted values and the expected values. The data utilized for this evaluation is sourced from the KU-HAR dataset. Figure 6 highlights the assessment of the proposed model’s performance on the KU-HAR dataset across varying hyper-parameter configurations outlined in Table 4. The analysis offers valuable insights into the impact of hyper-parameter variations on the overall effectiveness of the model when applied to the specified dataset. In Fig. 7, a visual representation offers an overview of the proposed framework to the KU-HAR dataset.

### Performance of proposed model using the WISDM dataset

A mobile application on Android smartphones was employed for WISDM dataset collection. Participants were instructed to place the smartphone in the front leg pocket and engage in five distinct supervised activities: walking, jogging, upstairs, downstairs, sitting, and standing. Throughout these activities, the accelerometer sensor recorded data at a consistent sampling rate of 20 Hz. The WISDM dataset comprises the raw time series data from the accelerometer. This dataset includes 5424 images of six different actions for 29 participants and further details are provided in Table 7.

**Table 7:** WISDM dataset description.

Class	#Images
Walking	2082
Jogging	1627
Upstairs	634
Downstairs	531
Sitting	309
Standing	241

Table 8 presents the attained accuracy, precision, and F-score during testing phases on subsets of the WISDM dataset. The highest accuracy observed on the testing subset, nearly reaching 94%, was achieved with a learning rate set at 0.0001, utilizing the SGD optimization technique. Precision and F-score consistently fall within the range of 94%, 93% under similar conditions. Through statistical analysis, it has been established that the average F-score and precision over multiple attempts approximate 93%, while the average accuracy stands at 94%. Median values indicate a sustained accuracy level, precision and F-score of around 93%. The standard deviation and variance at approximately 1.0 for accuracy, precision, and F-score. The data displays a moderate degree of dispersion from the mean values, indicating some variability in the dataset. Skewness values reveal a minor positive skewness of approximately − 0.2 for accuracy and F-score. The dataset’s range is approximately 3, representing the difference between the maximum and minimum values.

**Table 8:** Performance of the proposed model for WISDM dataset.

Optimizer	#Run	LR	Accuracy	Precision	F-score
SGD	Run (1)	0.01	92.73	94.02	93.16
	Run (2)	0.001	94.58	94.58	93.17
	Run (3)	0.0001	94.93	93.85	93.42
	Run (4)	0.00001	94.79	93.77	93.34
	Run (5)	0.000001	93.42	93.87	93.26
ADAM	Run (6)	0.01	92.77	93.14	92.51
	Run (7)	0.001	94.63	95.06	93.43
	Run (8)	0.0001	92.04	92.25	90.64
	Run (9)	0.00001	93.79	91.86	90.3
	Run (10)	0.000001	93.3	93.51	91.81
Statistical analysis					
Mean			93.6980	93.5910	92.5040
Standard error of mean			0.31882	0.30735	0.37498
Median			93.6050	93.8100	93.1650
Standard deviation			1.00820	0.97192	1.18578
Variance			1.016	0.945	1.406
Skewness			-0.228	-0.519	-1.196
Standard error of skewness			0.687	0.687	0.687
Range			2.89	3.20	3.13
Minimum			92.04	91.86	90.30
Maximum			94.93	95.06	93.43

**Fig. 8:**
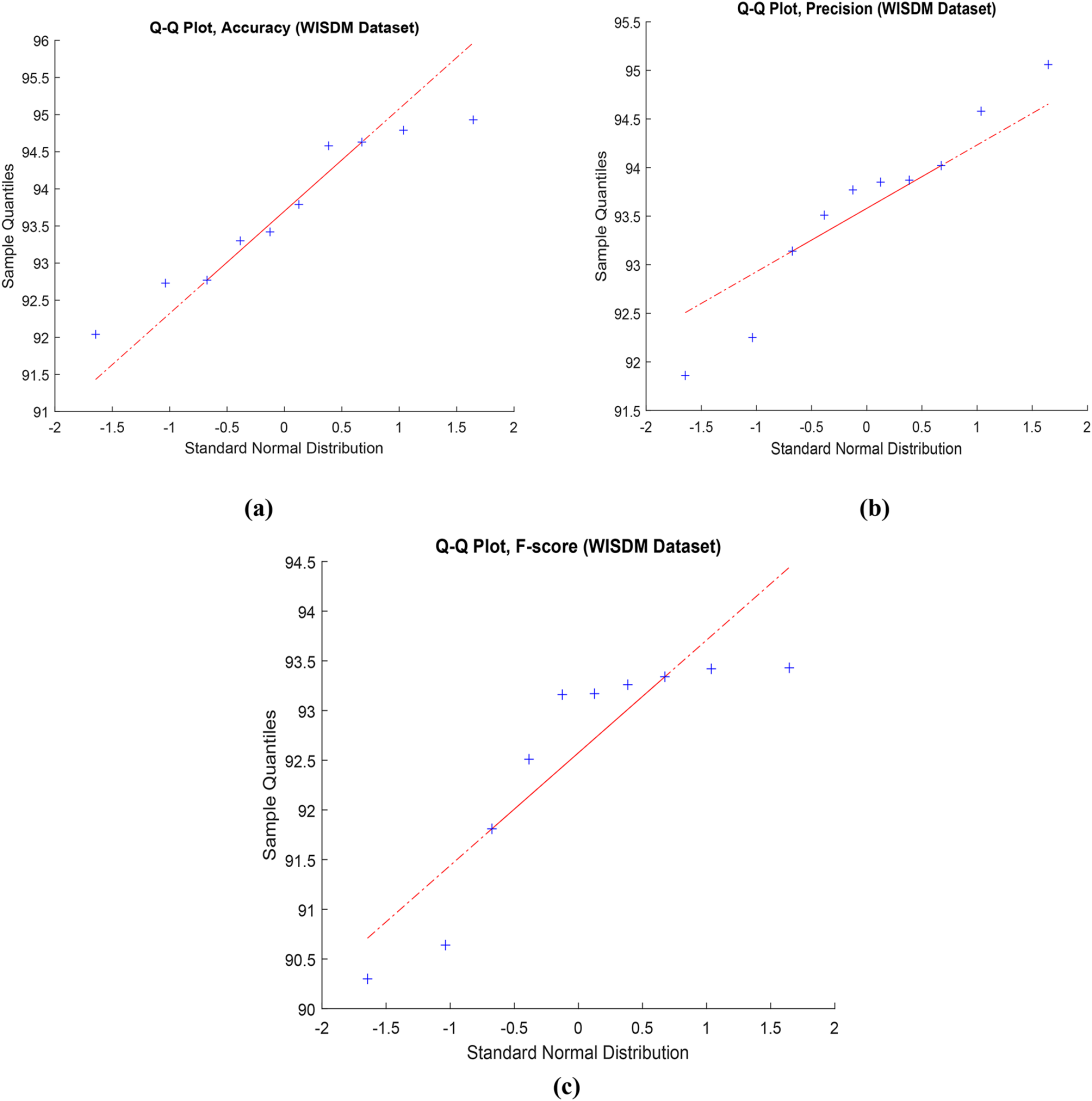
Q-Q Plot for accuracy, precision, and F-score in the proposed framework using WISDM dataset.

**Fig. 9:**
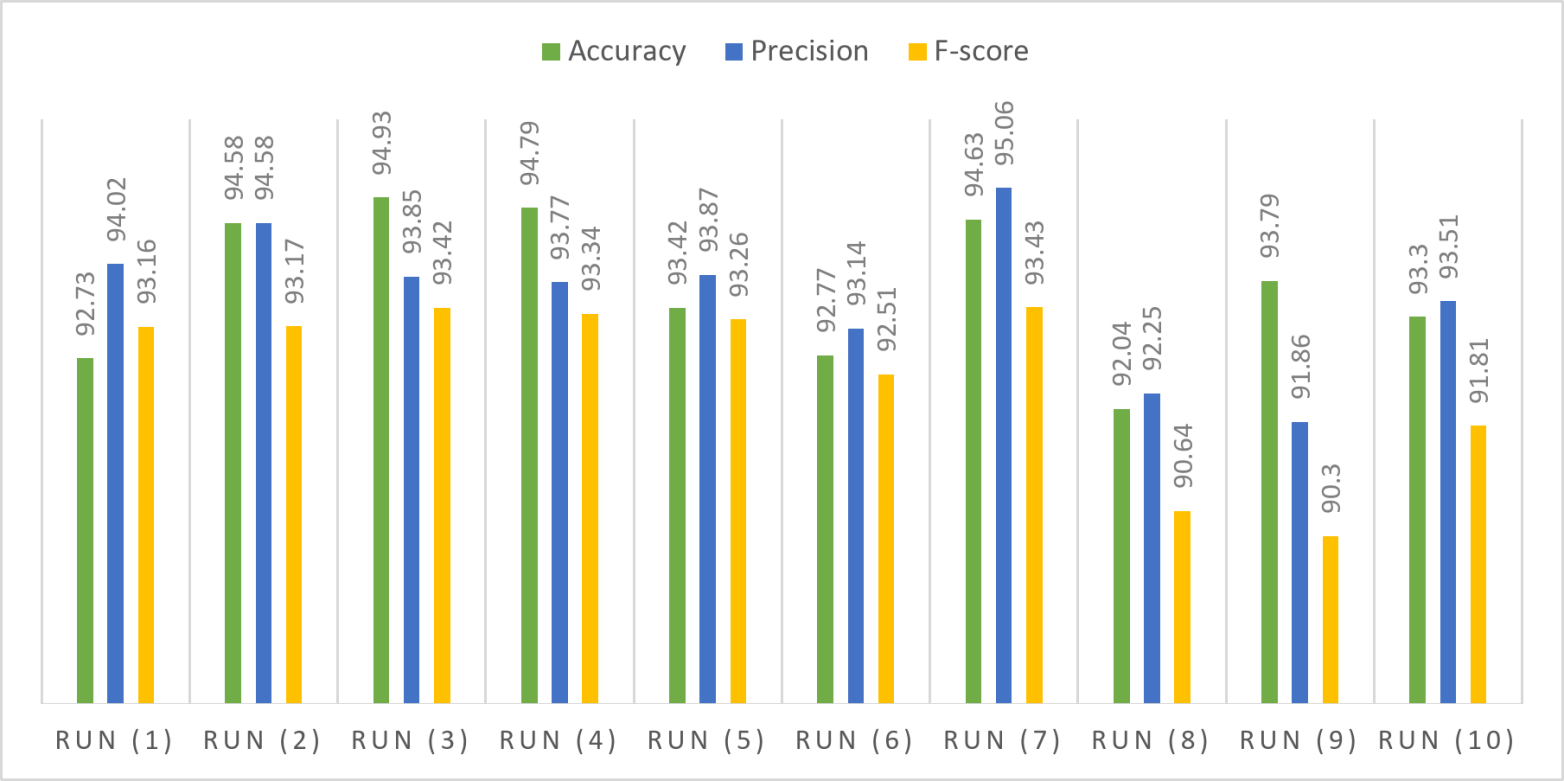
Performance assessment of the proposed model on WISDM dataset across varied hyper-parameters.

**Fig. 10:**
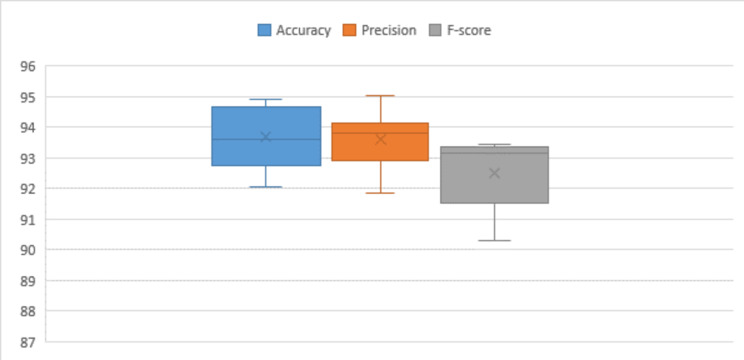
Visual representation of the proposed framework performance utilizing WISDM dataset.

Figure 9 used to compare the distribution of observed data with the expected distribution using Q-Q statistical technique. This approach, applied to key performance metrics such as accuracy, precision, and F-score, enables an assessment of the goodness of fit between the predicted values from the proposed framework and the expected values. The data utilized for this evaluation is sourced from the WISDM dataset. In Fig. 9, the evaluation of the proposed model’s performance on the WISDM dataset is presented across data configurations outlined in Table 6. This visual representation provides insights into the impact of hyper-parameter variations on the overall effectiveness of the model when applied to the specified dataset. Moving on to Fig. 10, a visual overview of the proposed framework’s application to the WISDM dataset is depicted. This visual representation offers a comprehensive overview of how the proposed framework operates on the WISDM dataset.

### Performance of proposed model using the HMDB51 dataset

The HMDB-51 dataset features exemplar actions, representing distinctive and characteristic activities captured in video clips. Notable actions within this dataset include fundamental human movements such as walking and running, activities related to daily life like eating and drinking, expressions of emotion like smiling, dynamic actions such as jumping, and skill-based activities like playing the guitar. Each video clip in the HMDB-51 dataset encapsulates a specific human action, providing a diverse representation of various activities. The HMDB51 dataset is composed of 6,766 video clips from 51 action categories, with each category containing at least 101 clips. Table 9 outlines accuracy, precision, and F-score on HMDB-51 dataset subsets during testing. The highest accuracy, nearly 98%, occurred with a 0.01 learning rate using ADAM optimization. Precision and F-score consistently stayed around 97%. The average accuracy is 97%, with F-score and precision averaging at 96%. Median values indicate sustained accuracy, precision, and F-score around 96%. Standard deviation and variance (0.6 for accuracy and F-score, 0.7 for precision) suggest moderate variability. Skewness is minor (0.7 for accuracy, -0.7 for precision, 0.05 for F-score), and the dataset’s range is approximately 2.

**Table 9:** Performance of the proposed model for HMDB51 dataset.

Optimizer	#Run	LR	Accuracy	Precision	F-score
SGD	Run (1)	0.01	96.63	95.58	95.82
	Run (2)	0.001	96.8	94.42	95.93
	Run (3)	0.0001	97.38	97.04	96.61
	Run (4)	0.00001	96.12	96.25	95.76
	Run (5)	0.000001	96.64	95.71	95.02
ADAM	Run (6)	0.01	96.22	96.33	95.7
	Run (7)	0.001	97.56	96.54	96.85
	Run (8)	0.0001	95.78	95.54	95.98
	Run (9)	0.00001	96.1	96.43	95
	Run (10)	0.000001	96.2	95.63	96.25
Statistical analysis					
Mean			96.5430	95.9470	95.8920
Standard error of mean			0.18249	0.23110	0.18815
Median			96.4250	95.9800	95.8750
Standard deviation			0.57708	0.73081	0.59499
Variance			0.333	0.534	0.354
Skewness			0.705	-0.716	-0.057
Standard error of skewness			0.687	0.687	0.687
Range			1.78	2.62	1.85
Minimum			95.78	94.42	95.00
Maximum			97.56	97.04	96.85

**Fig. 11:**
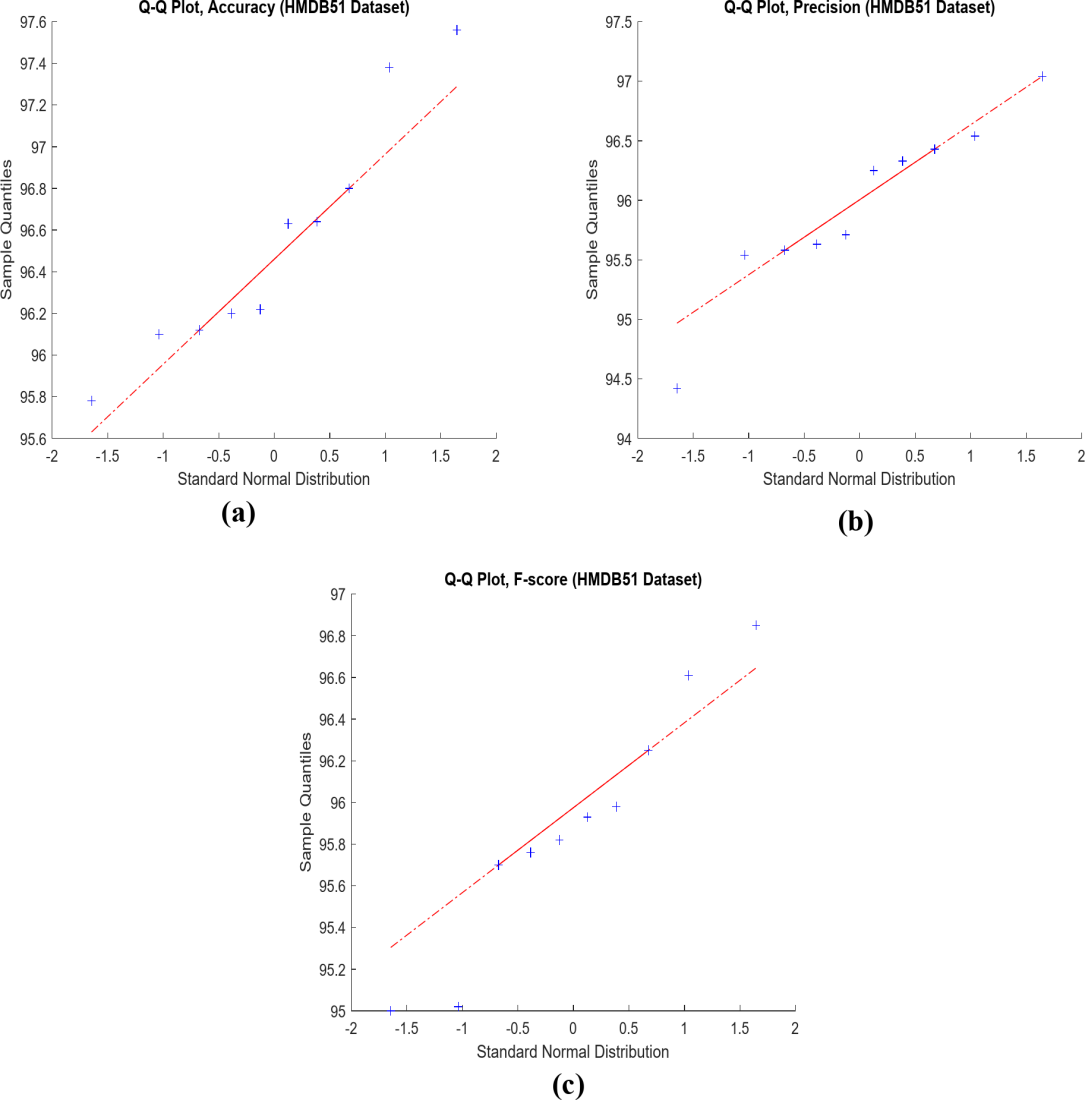
Q-Q Plot for accuracy, precision, and F-score of the proposed framework using HMDB-51 dataset.

**Fig. 12:**
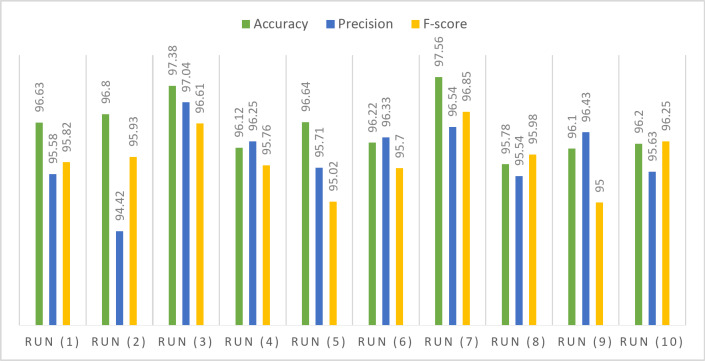
Performance assessment of the proposed model on HMDB-51 dataset across varied hyper-parameters.

**Fig. 13:**
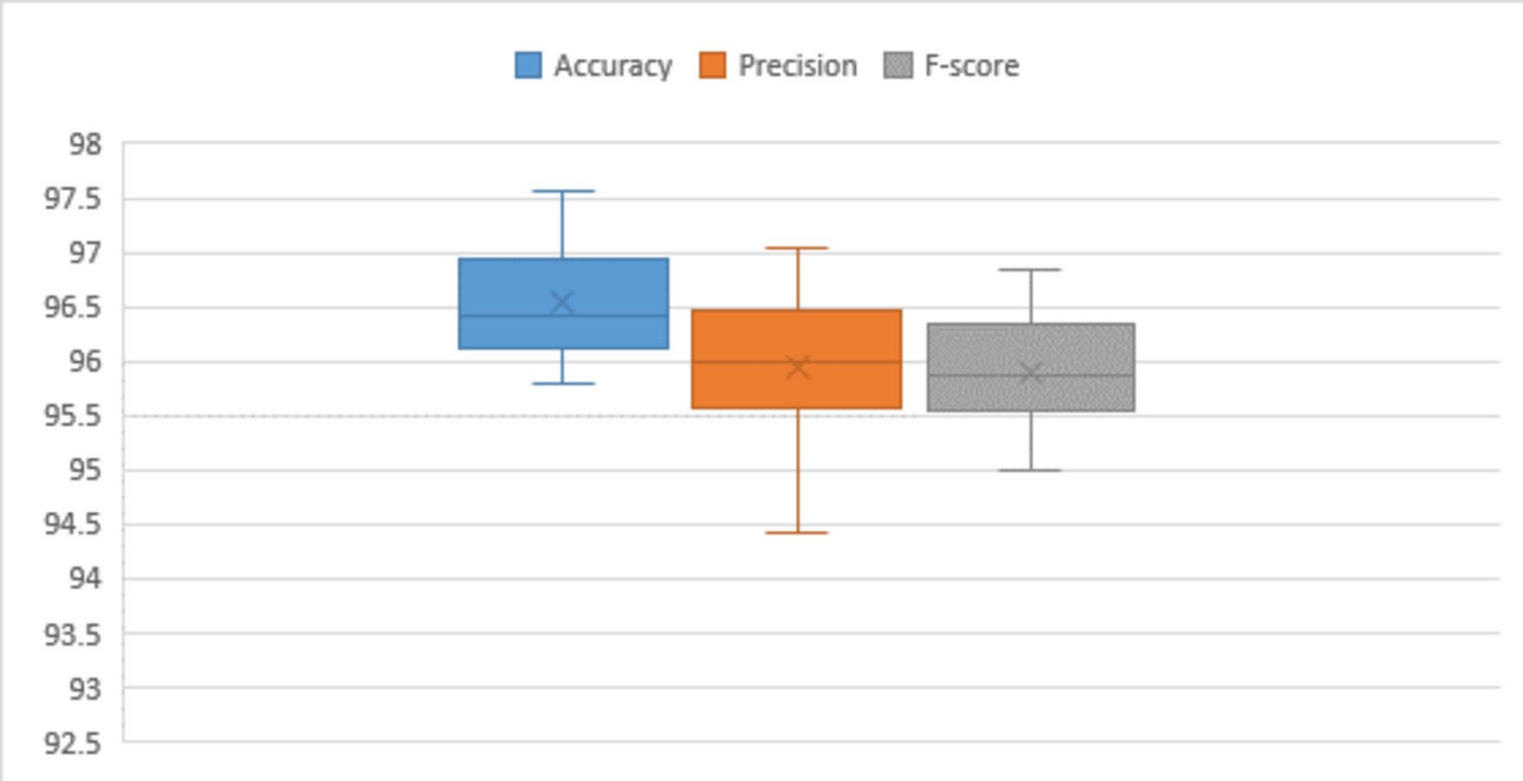
Visual representation of the proposed framework performance utilizing HMDB-51 Dataset.

In Fig. 11, the Q-Q statistical technique compares observed and expected distributions of key metrics (accuracy, precision, and F-score) using data from the HMDB51 dataset. Figure 12 illustrates the model’s performance on the HMDB51 dataset across different data configurations, highlighting the impact of hyper-parameter variations. Figure 13 provides a visual overview of the proposed framework’s application to the HMDB51 dataset, offering a comprehensive understanding of its operation on this specific dataset.

To enhance the evaluation of the proposed HARCNN framework, sensitivity results are provided in Table 10 for all datasets, accompanied by a detailed statistical analysis of the outcomes. The experiments performed using different optimizers (SGD and ADAM) and different values of learning rate (0.01, 0.001, 0.0001, 0.00001, and 0.000001). This inclusion aims to provide a comprehensive assessment of the HARCNN performance, highlighting its effectiveness across diverse scenarios. By incorporating these results, the evaluation becomes more robust, allowing for a clearer understanding of the model’s strengths and ensuring a well-rounded analysis of its capabilities. From the results, we notice that the best sensitivity for (UCI-HAR, KU-HAR, WISDM, and HMDB-51) datasets is achieved using (SGD optimizer & LR = 0.00001, SGD optimizer & LR = 0.00001, SGD optimizer & LR = 0.0001, and SGD optimizer & LR = 0.001) respectively.

**Table 10:** The sensitivity of the proposed framework for various datasets (UCI-HAR, KU-HAR, WISDM, AND HMDB-51), the experiments performed using different optimizers (SGD and ADAM) and different values of learning rate (0.01, 0.001, 0.0001, 0.00001, and 0.000001).

Optimizer	LR	UCI-HAR	KU-HAR	WISDM	HMDB-51
SGD	0.01	94.7954	95.59005	92.31559	96.06121
	0.001	92.12717	94.8101	91.80142	**97.48908**
	0.0001	94.75721	96.30229	**92.99392**	96.18379
	0.00001	**95.30357**	**97.33269**	92.91393	95.27496
	0.000001	93.89803	95.76177	92.65788	94.33988
ADAM	0.01	93.52296	95.81289	91.88847	95.07819
	0.001	90.07863	96.33189	91.85496	97.162
	0.0001	91.53877	95.56322	89.08523	96.42407
	0.00001	93.64995	96.74164	88.7921	93.61179
	0.000001	91.61911	96.69256	90.17071	96.87809
Statistical analysis					
Mean		93.129	96.0939	91.44742	95.85
Standard error of mean		0.54049	0.2305	0.4888516	0.39674
Median		93.58646	96.0575	91.8717	96.1225
Standard deviation		1.70918	0.7292	1.54588	1.2546
Variance		2.92	0.5317	2.38975	1.574
Skewness		−0.4721	−0.0151	-0.7769	-0.4349
Standard error of skewness		0.7746	0.7746	0.68704	0.687
Range		5.2249	2.5225	4.20181	3.8772
Minimum		90.0786	94.810	88.7921	93.6117
Maximum		95.3035	97.3326	92.9939	97.489

Significant values are in bold.

**Fig. 14:**
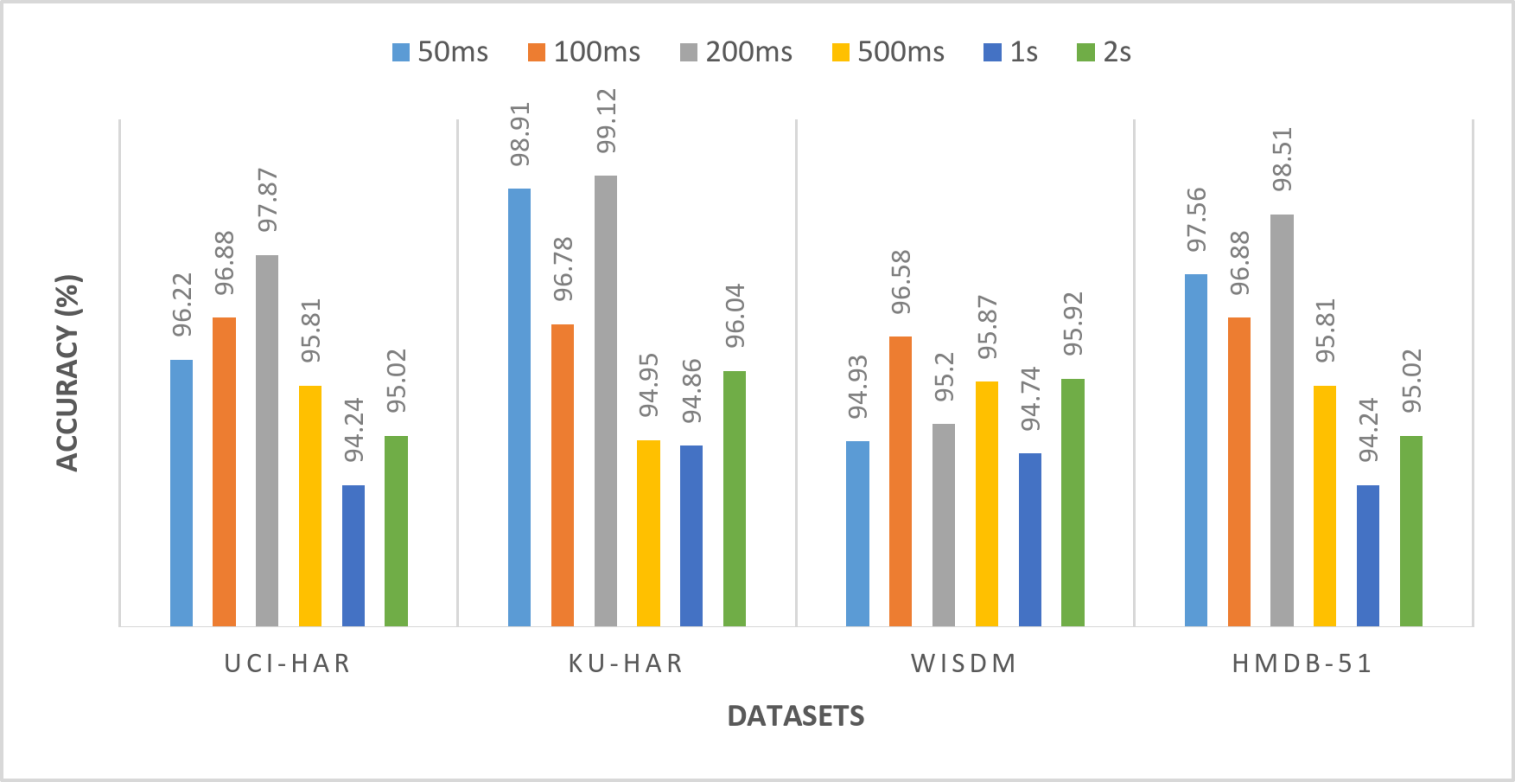
The accuracy of the proposed framework for various datasets (UCI-HAR, KU-HAR, WISDM, and HMDB-51), the experiments performed using different window sizes (50ms, 100ms, 200ms, 500ms, 1s, and 2s).

**Fig. 15:**
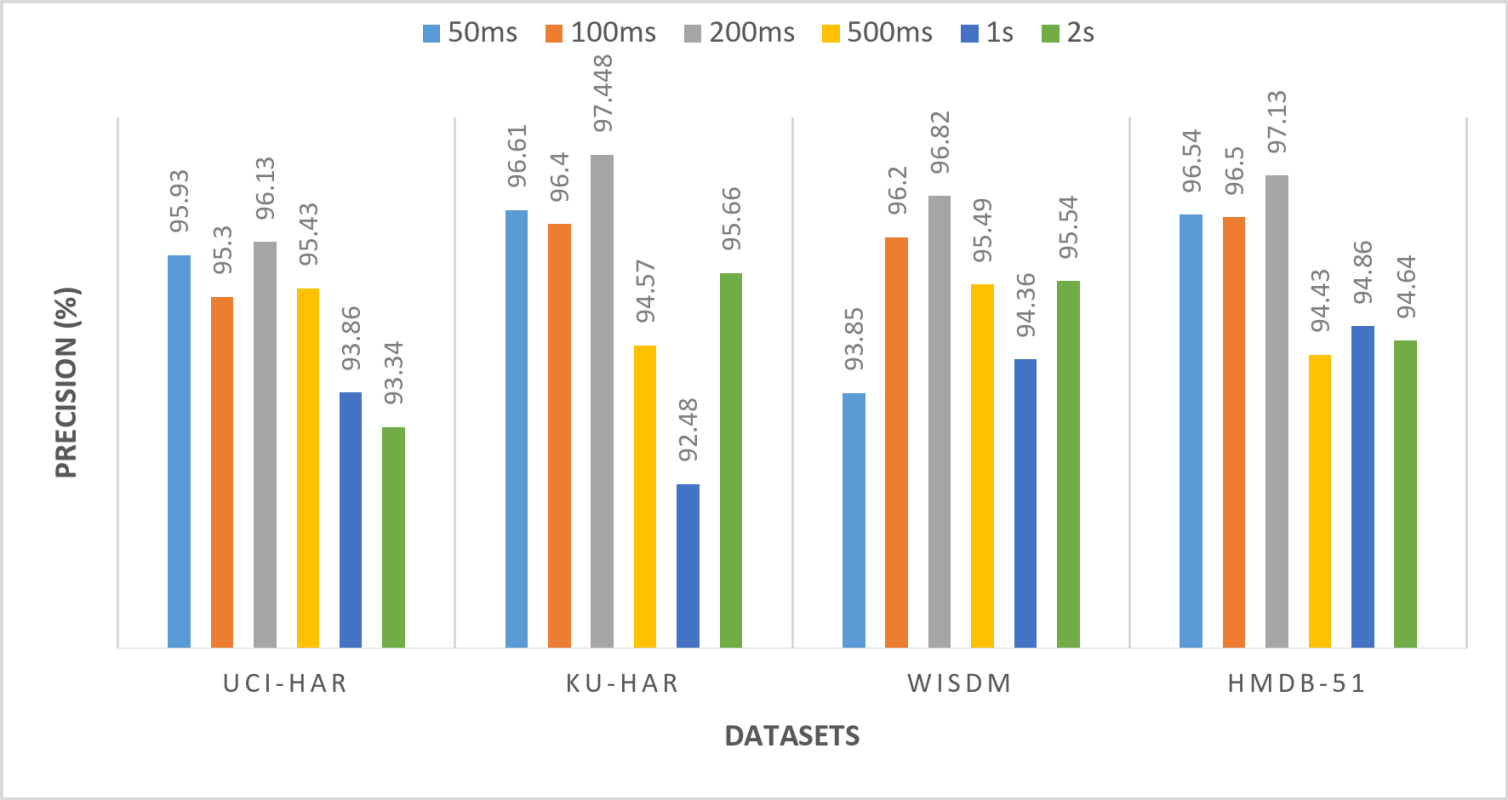
The precision of the proposed framework for various datasets (UCI-HAR, KU-HAR, WISDM, and HMDB-51), the experiments performed using different window sizes (50ms, 100ms, 200ms, 500ms, 1s, and 2s).

**Fig. 16:**
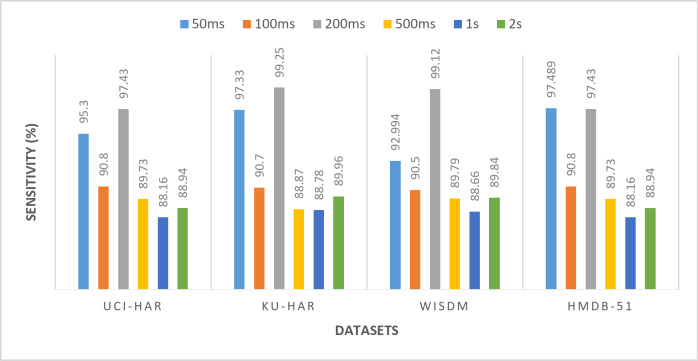
The sensitivity of the proposed framework for various datasets (UCI-HAR, KU-HAR, WISDM, and HMDB-51), the experiments performed using different window sizes (50ms, 100ms, 200ms, 500ms, 1s, and 2s).

**Fig. 17:**
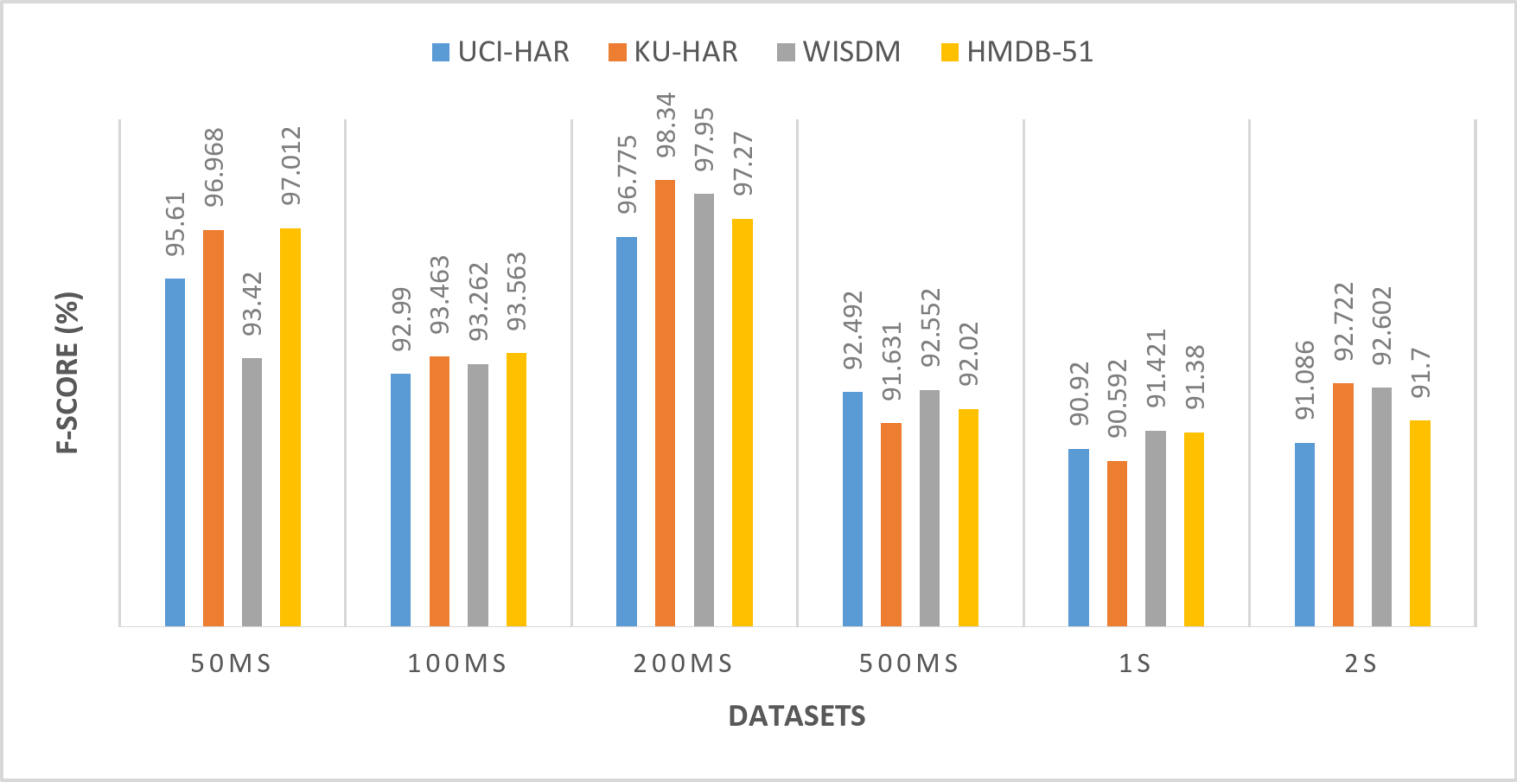
The F-score of the proposed framework for various datasets (UCI-HAR, KU-HAR, WISDM, and HMDB-51), the experiments performed using different window sizes (50ms, 100ms, 200ms, 500ms, 1s, and 2s).

All the previous results were performed using a window size of 50ms. To demonstrate the robustness of the proposed framework, experiments are conducted using different window sizes (100ms, 200ms, 500ms, 1s, and 2s) across UCI-HAR, KU-HAR, WISDM, AND HMDB-51 datasets. Varying the window size is a critical aspect of evaluating the generalizability and adaptability of the model, as it directly influences the segmentation of time-series data and the extraction of relevant features. Smaller windows capture fine-grained temporal dynamics, while larger windows provide a broader view of activity patterns. By testing the model with multiple window sizes, it becomes possible to assess its ability to perform well under varying data configurations, which is a crucial requirement for real-world applications where window sizes may differ based on the nature of the activity or the constraints of the data acquisition system. Tables 11, 12, 13 and 14 and Figs. 14, 15, 16 and 17 present the (accuracy, precision, sensitivity, and F-score) respectively. These results show the performance of the proposed framework for various datasets (UCI-HAR, KU-HAR, WISDM, and HMDB-51), the experiments performed using different window sizes (50ms, 100ms, 200ms, 500ms, 1s, and 2s). The results of these experiments reveal that the proposed method maintains high accuracy and reliability across diverse window sizes, underscoring its capability to adapt to different temporal granularities without significant performance degradation. This demonstrates that the method is not only effective but also robust, making it a strong candidate for deployment in varied HAR scenarios. Using window size of 200ms achieves the superior results.

**Table 11:** The accuracy of the proposed framework for various datasets (UCI-HAR, KU-HAR, WISDM, and HMDB-51), the experiments performed using different window sizes (50ms, 100ms, 200ms, 500ms, 1s, and 2s).

Window size	UCI-HAR	KU-HAR	WISDM	HMDB-51
50 ms	96.22	98.91	94.93	97.56
100 ms	96.88	96.78	**96.58**	96.88
200 ms	**97.87**	**99.12**	95.20	**98.51**
500 ms	95.81	94.95	95.87	95.81
1 s	94.24	94.86	94.74	94.24
2 s	95.02	96.04	95.92	95.02

Significant values are in bold.

**Table 12:** The precision of the proposed framework for various datasets (UCI-HAR, KU-HAR, WISDM, and HMDB-51), the experiments performed using different window sizes (50ms, 100ms, 200ms, 500ms, 1s, and 2s).

Window size	UCI-HAR	KU-HAR	WISDM	HMDB-51
50 ms	95.93	96.61	93.85	96.54
100 ms	95.30	96.40	96.2	96.5
200 ms	**96.13**	**97.448**	**96.82**	**97.13**
500 ms	95.43	94.57	95.49	94.43
1 s	93.86	92.48	94.36	94.86
2 s	93.34	95.66	95.54	94.64

Significant values are in bold.

**Table 13:** The sensitivity of the proposed framework for various datasets (UCI-HAR, KU-HAR, WISDM, AND HMDB-51), the experiments performed using different window sizes (50ms, 100ms, 200ms, 500ms, 1s, and 2s).

Window size	UCI-HAR	KU-HAR	WISDM	HMDB-51
50 ms	95.3	97.33	92.994	97.489
100 ms	90.8	90.7	90.5	90.8
200 ms	**97.43**	**99.25**	**99.12**	**97.43**
500 ms	89.73	88.87	89.79	89.73
1 s	88.16	88.78	88.66	88.16
2 s	88.94	89.96	89.84	88.94

Significant values are in bold.

**Table 14:** The F-score of the proposed framework for various datasets (UCI-HAR, KU-HAR, WISDM, AND HMDB-51), the experiments performed using different window sizes (50ms, 100ms, 200ms, 500ms, 1s, and 2s).

Window size	UCI-HAR	KU-HAR	WISDM	HMDB-51
50 ms	95.61	96.968	93.42	97.012
100 ms	92.99	93.463	93.262	93.563
200 ms	**96.775**	**98.340**	**97.95**	**97.27**
500 ms	92.492	91.631	92.552	92.02
1 s	90.920	90.592	91.421	91.38
2 s	91.086	92.722	92.602	91.7

Significant values are in bold.

### Comparative analysis with recent methods

We have compared the performance of our proposed HARCNN model with a number of pre-trained CNN models and various state-of-the-art classification techniques. Table 15 provides the comparison. As evident from the table, concerning the KU-HAR dataset, our method surpassed the performance of all the recent studies. It achieved an accuracy of 99.12%, which documented a higher accuracy and F-score than other works. In the context of the UCI HAR dataset, our proposed method stands out as superior when compared to numerous other studies that have been investigated. For the WISDM dataset, our approach demonstrates a 2.69% improvement in accuracy and 4.7% improvement in F-score. Finally, the results of the HARCNN model for HMDB-51 images proves the success of our algorithm. The proposed model demonstrates an impressive accuracy of 98.51%, along with F-score reaching 97.27%.

**Table 15:** Comparison between the proposed HARCNN framework and other traditional pre-trained and state-of-the-art CNNs in terms of accuracy and F-score.

Method	Dataset	Accuracy	F-score
^9^	KU-HAR	96.86	96.92
^29^		89.67	87.59
^28^		96.67	96.41
^30^		88.53	88.52
^31^		93.18	94.25
VGG19		92.97	93.13
NasnetMobile		93.94	94.37
ShuffleNet		94.91	95.28
MobileNetv2		93.48	93.84
GoogleNet		93.37	93.92
Proposed		99.12	98.340
^9^	UCI-HAR	93.48	92.88
CNN^32^		92.93	92.71
Res-LSTM^33^		91.5	91.6
Stacked-LSTM^34^		92.75	93.13
CNN-LSTM^35^		92.67	92.13
Bidir-LSTM ^36^		92.91	92.67
Residual-BiLSTM^33^		93.5	93.6
LSTM-CNN^37^		95.22	95.78
CNN-GRU^38^		93.65	96.2
CNN-GRU^39^		94.54	94.58
CNN-LSTM^30^		94.76	94.8
HS-Resnet^21^		97.38	94.10
VGG19		90.88	91.76
NasnetMobile		91.85	93
ShuffleNet		92.82	93.91
MobileNetv2		91.39	92.47
GoogleNet		91.28	92.55
Proposed		97.87	96.775
^9^	WISDM	93.89	92.14
HS-Resnet^21^		93.20	93.24
VGG19		90.65	91.1
NasnetMobile		91.62	92.34
ShuffleNet		92.59	93.25
MobileNetv2		91.16	91.81
GoogleNet		91.05	91.89
Proposed		96.58	97.95
^36^	HMDB51	96.7	95.11
CNN ^22^		96.58	96.31
VGG19		95.95	94.17
NasnetMobile		95.92	94.41
ShuffleNet		96.89	96.32
MobileNetv2		95.46	95.88
GoogleNet		96.35	95.96
Proposed		98.51	97.27

## Conclusion

The proposed HARCNN framework marks a significant advancement in the domain of Human Activity Recognition (HAR), addressing longstanding challenges associated with noisy sensor data and achieving state-of-the-art performance metrics. By employing a custom-designed Convolutional Neural Network (CNN) architecture with 10 meticulously crafted convolutional blocks, the HARCNN model demonstrates exceptional ability to extract hierarchical spatial and temporal features from raw accelerometer and gyroscope data. The integration of depth concatenation for feature fusion across multiple abstraction levels further amplifies the model’s capacity to capture intricate motion patterns and dependencies inherent in human activities. Rigorous experimentation using diverse datasets, including UCI-HAR, KU-HAR, WISDM, and HMDB51, validates the robustness and adaptability of the proposed model. The achieved accuracies of 97.87%, 99.12%, 96.58%, and 98.51% across these datasets highlight its efficacy in distinguishing between human activities with remarkable precision. Notably, the model demonstrates its resilience to varying temporal granularities, as evidenced by consistent performance across window sizes ranging from 50ms to 2s, with optimal results obtained at 200ms. This adaptability underscores the framework’s potential for deployment in real-world scenarios where sensor sampling rates and temporal resolutions can vary. Compared to traditional pre-trained CNNs and other state-of-the-art methods, HARCNN consistently outperforms in terms of accuracy, precision, sensitivity, and f-score, minimizing false positives and negatives—crucial metrics for practical applications. The strategic architectural choices, such as the inclusion of batch normalization and ReLU activation functions, not only enhance learning stability but also contribute to the model’s superior generalization capabilities. This study highlights the transformative potential of leveraging advanced CNN-based architectures for HAR. The proposed framework’s design principles, including modular convolutional blocks and sophisticated feature fusion strategies, establish a robust foundation for future research. It sets a precedent for exploring more dynamic recognition systems, such as those capable of online learning and adaptive tuning in response to changing environmental or user-specific conditions.

HARCNN model is designed for efficient inference with acceptable latency on mobile devices. Computation efficiency is guaranteed by the model’s architecture, which consists of ten convolution blocks with hierarchical feature fusion. By utilizing hardware accelerators like NPUs (Neural Processing Units) and GPUs (Graphics Processing Units), it may also be tailored for mobile deployment using frameworks like TensorFlow Lite, ONNX Runtime Mobile, and Core ML. The suggested model can be quantized to decrease memory usage and speed up execution. Techniques for quantization are significantly cut down on execution time and memory utilization. The model’s ability to process varying temporal window sizes (e.g., 100ms–200ms) allows real-time inference while maintaining high accuracy (e.g., 99.12% on KU-HAR and 97.87% on UCI-HAR), making it well-suited for mobile applications in healthcare, sports, and human-computer interaction.

In conclusion, the HARCNN model is a versatile and efficient solution that bridges the gap between theoretical advancements and practical applications in HAR. Its superior performance across datasets and adaptability to temporal variations make it a promising candidate for integration into healthcare monitoring systems, fitness trackers, and human-computer interaction platforms. Future research may explore extending this framework to accommodate multimodal sensor data, real-time processing, and application-specific optimizations to further expand its utility in diverse HAR scenarios.

## Data Availability

The dataset generated and analyzed during the current study are available at https://www.kaggle.com/datasets/meetnagadia/human-action-recognition-har-dataset All other data underlying the results are available as part of the article and no additional source data are required.
